# Association between frailty syndrome and sedentary behavior among community-dwelling older adults in the Amazon region: a cross-sectional study

**DOI:** 10.1590/1516-3180.2020.0546.r1.14122020

**Published:** 2021-03-12

**Authors:** Izabelle Santos dos Santos, Caroline de Fátima Ribeiro Silva, Daniela Gonçalves Ohara, Areolino Pena Matos, Ana Carolina Pereira Nunes Pinto, Maycon Sousa Pegorari

**Affiliations:** I Undergraduate Physiotherapy Student, Universidade Federal do Amapá (UNIFAP), Macapá (AP) Brazil.; II PT. Physiotherapist and Postgraduate Student on Health Science Course, Universidade Federal do Amapá (UNIFAP), Macapá (AP) Brazil.; III PhD. Physiotherapist and Adjunct Professor, Physiotherapy Course, Universidade Federal do Amapá (UNIFAP), Macapá (AP) Brazil.; IV PhD. Physiotherapist and Adjunct Professor, Physiotherapy Course, Universidade Federal do Amapá (UNIFAP), Macapá (AP) Brazil.; V PhD. Physiotherapist and Adjunct Professor, Physiotherapy Course, Universidade Federal do Amapá (UNIFAP), Macapá (AP) Brazil.; VI PhD. Physiotherapist and Adjunct Professor, Physiotherapy Course, Universidade Federal do Amapá (UNIFAP), Macapá (AP) Brazil.

**Keywords:** Aged, Health services for the aged, Sedentary behavior, Frail elderly, Older adults, Health of the elderly, Physical inactivity

## Abstract

**BACKGROUND:**

Existence of an association between sedentary behavior and frailty among older adults has been suggested. However, there is a lack of studies conducted in Brazil, especially in areas of the Amazon region.

**OBJECTIVE:**

To analyze the association between frailty syndrome and sedentary behavior among community-dwelling older adults.

**DESIGN AND SETTING:**

Cross-sectional study carried out in Macapá, state of Amapá, Brazil.

**METHODS:**

Frailty status was assessed using Fried's frailty phenotype, and sedentary behavior was evaluated using two questions concerning time spent in a seated position, from the International Physical Activity Questionnaire (IPAQ). A multinomial logistic regression model was used to verify the association between frailty syndrome and sedentary behavior.

**RESULTS:**

The final study sample was made up of 411 older adults with a mean age of 70.14 ± 7.25 years and an average daily duration of sedentary behavior of 2.86 ± 2.53 hours. The prevalence of non-frailty was 28.7%, prevalence of pre-frailty was 58.4% and prevalence of frailty was 12.9%. The adjusted analysis showed that there were independent associations between sedentary behavior and pre-frailty (odds ratio, OR = 1.18; 95% confidence interval, CI: 1.03-1.34) and between sedentary behavior and frailty (OR = 1.20; 95% CI: 1.02-1.40).

**CONCLUSION:**

Frailty and pre-frailty status were associated with sedentary behavior among community-dwelling older adults.

## INTRODUCTION

Over recent decades, the older adult population has dramatically increased worldwide.^1^ This global phenomenon provides a challenge for healthcare systems,^2^ and the World Health Organization has emphasized that achieving successful aging is currently a major concern.^3^ Therefore, reducing the risks of sarcopenia, frailty and non-communicable diseases (NCDs) is a key goal for both individuals and policymakers.^3^

Diminution of sarcopenia (i.e. age-related loss of muscle mass and strength) could play a central role in reducing these risks, given that presence of sarcopenia can lead to reduced physical performance and impaired ability to perform activities of daily living, therefore increasing the risk of being frail.^4^ Frailty (i.e. a state of vulnerability to stressors due to cumulative decline of physiological systems over the course of an individual's life) is associated with increased risk of falls, fractures, hospitalizations, iatrogenic complications and death.^5^^–^^7^ In parallel with research on sarcopenia and frailty, sedentary behavior has gained prominence in the literature as a risk factor for NCDs.^8^

NCDs are the leading causes of death worldwide, with a disproportionate burden in low and middle-income countries.^9^ Furthermore, a previous systematic review demonstrated that there was a clear dose-response relationship between physical activity and all-cause mortality among middle-aged and older adults.^10^ Importantly, sedentary behavior has also been associated with sarcopenia^11^ and frailty^12^ among older adults.

Several studies have provided robust evidence on the relationship between sedentary behavior, physical activity and sarcopenia.^11^^,^^13^^–^^18^ However, the association between sedentary behavior, physical activity and frailty has been less investigated. A recent systematic review investigated the association of sedentary behavior and frailty and highlighted the heterogeneity of samples included across studies, which may limit the generalizability of the results.^12^

It is noteworthy that there is a lack of studies conducted in the Amazon region, a place with great ethnocultural diversity and different socioeconomic characteristics. The diversity of this region may influence behaviors and health outcomes.^19^

Investigating this relationship can provide evidence for future healthcare policy strategies for elderly populations and for clinical decision-making processes.

## OBJECTIVE

The aim of this study was to analyze the association between frailty syndrome and sedentary behavior among community-dwelling older adults. The hypothesis of this study was that sedentary behavior would be positively associated with frailty syndrome among older adults.

## METHODS

### Design and sample

This cross-sectional study was conducted in the urban area of Macapá, state of Amapá, Brazil. The study was approved (protocol no. 1.738.671; dated September 21, 2016) by the local human research ethics committee, and all participants signed an informed consent statement before commencing their study participation. Some results from this study have been previously reported.^20^^–^^22^

The urban population of Macapá was defined using multi-stage sampling with random selection of conglomerates. For the sample size calculation, the health problem was assumed to have a prevalence of 50% among the urban population of 19,955 older adults. With an accuracy of 5% and a confidence interval of 95%, a minimum sample of 377 individuals was found to be needed, to reach a representative sample of this population.

Adults aged 60 years or over who were able to walk, with or without walking assistance devices, were included. The exclusion criteria included: being lost to follow-up after three attempts, being institutionalized or hospitalized, having neurological diseases that made it impossible to do evaluations or presenting cognitive decline. The validated Brazilian version of the Mini-Mental State Examination was used to screen participants for cognitive decline.^23^ A total of 443 older adults were recruited and assessed, of whom 27 were excluded because they showed cognitive decline, and another 5 were excluded for other reasons (such as incomplete data). After considering the eligibility and loss criteria, 411 community-dwelling older adults were finally included in this study.^22^

### Data collection

#### Frailty syndrome (dependent variable)

Frailty was assessed using the frailty phenotype proposed by Fried et al.,^24^ which is divided into five criteria.

The first criterion, (i) *Loss of muscle strength*, was assessed by means of a handgrip strength test, using a manual hydraulic dynamometer, and this was done in line with the recommendations of the American Society of Hand Therapists.^25^ The mean of three measurements was used for the analysis, segmented with the cutoff values proposed by Fried et al.^24^

The second criterion, (ii) *Self-reported fatigue*, was evaluated using two items (7^th^ and 20^th^) of the Brazilian version of the Center for Epidemiological Studies (CES-D) scale.^26^ Participants who answered “2” or “3” to either of these items fulfilled the self-reported fatigue criterion.

The third criterion, (iii) *Low level of physical activity* was evaluated using the elderly-adapted long version of the International Physical Activity Questionnaire (IPAQ).^27^ Participants who spent zero to 149 minutes per week on physical activities were classified as insufficiently active, as suggested by the American Heart Association and the College of Sports Medicine.^28^ Those who spent 150 minutes or more on physical activities were considered sufficiently active.

The fourth criterion, (iv) *Slowed walking speed*, was defined as the time taken for the participant to walk the middle 4.6 meters of an 8.6-meter course, at his or her usual pace. The first two meters (acceleration) and the last two meters (deceleration) were excluded. The mean of three measurements was recorded for the analysis.

Lastly, (v) *Unintentional weight loss* was assessed through the question: “In the last year, have you lost more than 4.5 kg unintentionally (i.e. not due to dieting or exercise)?”

Individuals fulfilling three or more of these five components were classified as frail. Those who fulfilled one or two components were considered pre-frail, and those who fulfilled none of these criteria were classified as non-frail.^24^

### Sedentary behavior (independent variable)

Sedentary behavior was the independent variable. The length of time exposed to sedentary behavior was evaluated through questions on the amount of time spent in a seated position on a usual weekday and on a usual weekend day (minutes/day). This was assessed using questions from IPAQ,^29^ as validated for the elderly Brazilian population.^30^^,^^31^ A weighted average, calculated as [(weekday x 5) + (weekend day x 2)]/7, was considered for the analysis.

### Adjustment variables

The exploratory and adjustment variables were the following: (i) socioeconomic: age, gender, education, living arrangement and marital status; (ii) physical health: health perception, smoking and hospitalization in the last year (yes/no); and (iii) functional capacity. Functional capacity was measured using the Katz index^32^ and the Lawton and Brody scale,^33^ which assess functional impairment with regard to basic and instrumental activities of daily living, respectively.

### Data analysis

The descriptive statistics calculated included means, standard deviations, absolute numbers and percentages. To verify associations between the categorical variables, the chi-square test was applied; and, between numerical variables, one-way analysis of variance (ANOVA) with post-hoc Dunnett T3, for multiple comparisons among groups (P < 0.05). A multinomial logistic regression model, adjusted for socioeconomic, clinical and health characteristics, was run to examine the association between frailty syndrome and sedentary behavior. Odds ratios (OR) with 95% confidence interval (CI) and a significance level of 5% (P < 0.05) were used. All the data were analyzed using version 25.0 of the Statistical Package for the Social Sciences (SPSS) software (New Orchard Road, Armonk, New York, United States).

## RESULTS

Four hundred and eleven older adults with a mean age of 70.14 ± 7.25 years were evaluated. Women comprised 66.4% of the sample. The length of time spent on sedentary behavior was 2.86 ± 2.53 hours. Only 12.9% (n = 53) of the participants were classified as frail, while 58.4% (n = 240) were classified as pre-frail and 28.7% (n = 118) as non-frail. Frailty syndrome was associated with age, education, marital status, health perception, hospitalization in the last year and functional dependence regarding basic and instrumental activities of daily living (P < 0.05) (**Table 1**)

**Table 1:** Socioeconomic, clinical and health characteristics of community-dwelling older adults. Macapá, Amapá, Brazil, 2017 (n = 411)

		Frailty syndrome				Total sample (n=411)
Variables		Frail53 (12.9%)	Pre-frail240 (58.4%)	Non-frail118 (28.7%)	P	
**Age (years)**		74 ± 8.49	70.64 ± 7.26	67.41 ± 5.44	< 0.001*	70.14 ± 7.25
**Sex**						
	Male	13 (24.5%)	77 (32.1%)	48 (40.7%)	0.088	138 (33.6%)
	Female	40 (75.5%)	163 (67.9%)	70 (59.3%)		273 (66.4%)
**Education (years)**		4.43 ± 4.37	5.61 ± 5.21	6.78 ± 5.55	0.010**	5.79 ± 5.25
Marital status						
	With partner	49 (41.5%)	140 (58.3%)	31 (58.5%)	0.008	220 (53.5%)
	Without partner	69 (58.5%)	100 (41.7%)	22 (41.5%)		191 (46.5%)
**Living arrangement**						
	Alone	2 (3.8%)	15 (6.2%)	11 (9.3%)	0.357	28 (6.8%)
	Accompanied	51 (96.2%)	225 (93.8%)	107 (90.7%)		383 (93.2%)
**Health perception**						
	Positive	53 (44.9%)	63 (26.3%)	8 (15.4%)	< 0.001	124 (30.2%)
	Negative	65 (55.1%)	177 (73.8%)	44 (84.6%)		286 (69.6%)
**Smoking habit**						
	Yes	10 (8.5%)	25 (11.7%)	1 (1.9%)	0.081	39 (9.5%)
	No	118 (91.5%)	212 (88.3%)	52 (98.1%)		372 (90.5%)
**Hospitalization in the last year**						
	Yes	10 (8.5%)	35 (14.6%)	13 (24.5%)	0.019	58 (14.1%)
	No	108 (91.5%)	205 (85.4%)	40 (75.5%)		353 (85.9%)
**BADL**						
	Dependent	8 (15.1%)	18 (7.5%)	4 (3.4%)	0.024	30 (7.3%)
	Independent	45 (84.9%)	222 (92.5%)	114 (96.6%)		381 (92.7%)
**IADL**						
	Dependent	43 (81.1%)	175 (72.9%)	68 (57.6%)	0.002	286 (69.6%)
	Independent	10 (18.9%)	65 (27.1%)	50 (42.4%)		125 (30.4%)
**Sedentary behavior (hours)**		3.28 ± 2.77	3.01 ± 2.77	2.40 ± 1.74	0.038***	2.86 ± 2.53

Data are reported as n = number of subjects; mean ± standard deviation; P < 0.05; BADL = basic activities of daily living; IADL = instrumental activities of daily living; Multiple comparisons between groups: significant differences (P < 0.05) were observed between the three groups.

* Non-frail ≠ Pre-frail ≠ Frail

** Non-frail ≠ Frail

*** Non-frail ≠ Pre-frail.

The adjusted analysis showed that there were independent positive associations between sedentary behavior and frailty (OR = 1.20; 95% CI: 1.02-1.40) and between sedentary behavior and pre-frailty (OR = 1.18: 95% CI: 1.03-1.34) (**Table 2**).

**Table 2:** Association between frailty syndrome and sedentary behavior, controlled for socioeconomic, clinical and health characteristics. Macapá, Amapá, Brazil, 2017 (n = 411)

		Frailty syndrome*					
Variables			Pre-frail			Frail	
		OR	95% CI	P	OR	95% CI	P
**Age (years)**		1.06	1.02-1.10	0.003	1.11	1.05-1.17	< 0.001
**Sex**							
	Male	1.95	1.17-3.26	0.755	1.66	0.70-3.88	0.243
	Female		1			1	
**Education (years)**		0.98	0.94-1.03	0.556	0.96	0.88-1.03	0.317
**Marital status**							
	With partner		1			1	
	Without partner	0.50	0.29-0.85	0.011	0.62	0.28-1.37	0.243
**Living arrangement**							
	Alone	1.72	0.67-4.41	0.255	1.54	0.27-8.56	0.619
	Accompanied		1			1	
**Health perception**							
	Positive		1			1	
	Negative	1.95	1.17-3.26	0.010	3.24	1.31-8.02	0.011
**Smoking habit**							
	Yes	2.19	0.94-5.07	0.067	0.41	0.04-3.52	0.418
	No		1			1	
**Hospitalization in the last year**							
	Yes	2.02	0.90-4.51	0.085	3.42	1.23-9.47	0.011
	No		1			1	
**BADL**							
	Dependent	2.02	0.61-6.67	0.249	3.01	0.73-12.46	0.127
	Independent		1			1	
**IADL**							
	Dependent	1.46	0.87-2.47	0.149	1.55	0.65-3.72	0.321
	Independent		1			1	
**Sedentary behavior (hours)**		1.18	1.03-1.34	0.013	1.20	1.02-1.40	0.023

Data are reported as n = number of subjects; mean ± standard deviation; OR = odds ratio; 95% CI = 95% confidence interval; P < 0.05; BADL = basic activities of daily living: IADL = instrumental activities of daily living; 1 = reference category; “Non-frail = reference category; Adjusted for age, gender, education, living arrangement, marital status, health perception, smoking, hospitalization in the last year and functional impairment regarding basic and instrumental activities of daily living.

## DISCUSSION

The findings from this study conducted using a representative sample of older adults differ from those in previous studies conducted in Brazil and elsewhere around the world, in community-dwelling and hospitalized elderly populations.^34^^–^^38^ We found shorter exposure to sedentary behavior than in previous studies and independent associations between sedentary behavior and both frailty and pre-frailty. While we found that community-dwelling older adults were spending nearly 2.88 hours engaged in sedentary behavior, previous studies have found that older adults engage in sedentary behavior for an average of 6.1^37^ to 8.5 hours^34^ per day. The exposure to sedentary behavior in the present study was also shorter than the exposure reported in other Brazilian studies. Da Silva et al. analyzed 285 women and 172 men between 60 and 97 years old, and identified that the an average length of time that they were spending on sedentary behavior was nearly 7 hours a day.^37^

It should be noted that previous studies have demonstrated that respondents may overestimate their physical activity levels when using self-reported instruments.^39^^,^^40^ Nevertheless, previous Brazilian studies that used self-reported instruments also found higher values for exposure to sedentary behavior than we found in our present study.^37^

Our hypothesis for the difference regarding the time spent on sedentary behavior is that the population of this study has specific sociocultural conditions. It is known that older women are usually more physically active than older men,^41^^,^^42^ as older women tend to spend more time on physical activities, such as doing housework and taking care of their grandchildren.^43^ Moreover, domestic physical activity accounts for a significant proportion of self-reported daily physical activity, particularly among females.^43^ Since most of our sample was composed of women, and women in the north of Brazil often do most of the housework,^19^ this may explain why we found lower values for exposure to sedentary behavior than in previous studies.

Another important factor relates to the place of residence. A previous study conducted among older adults in the Amazon region found that these individuals engaged in higher levels of physical activity than what had been found in previous studies.^19^ These results suggest that the physical activity level of older adults and, consequently, the aging process, is influenced by where they have resided over their lives.^19^ Further studies may clarify the influence of housing conditions on the exposure to sedentary behavior and physical activity levels among older adults in the Amazon region.

The prevalence of frailty (12.9%) in the present study was also lower than what was reported through a systematic review with meta-analysis on studies conducted in countries in Latin America and the Caribbean, in which it was found that 19.6% of the older adults were frail.^44^ One likely explanation for divergences in the prevalence of frailty may lie in the conceptual and operational definitions used for identification of frailty, along with the locoregional specificities of the study sample.^45^ Importantly, a previous study also found lower prevalence of frailty (9.4%) among older adults in the Amazon region than among those in other Brazilian cities. The authors of that study highlighted that the influence that Amazonian culture and environment have on lifestyle over the course of life may have a protective effect on health outcomes in later life.^46^

In line with our hypothesis, sedentary behavior was associated with both pre-frailty and frailty. In agreement with our findings, Silva Coqueiro et al.,^47^ Da Silva et al.^37^ and Da Silva et al.^48^ found associations between frailty and sedentary behavior among older adults. However, our study was the first Brazilian study to also identify an association between pre-frailty and sedentary behavior, even after adjusting for other variables. This result is especially important, since it indicates that as the exposure to sedentary behavior rises, the level of frailty increases. In line with this hypothesis, a previous study found that the effect of sedentary behavior on mortality varied according to the level of frailty, such that participants with the highest frailty level experienced the greatest adverse impact on mortality rates.^49^

The fact that most of our sample was found to be pre-frail (58.4%) deserves special attention, considering that this group is more likely to progress to frailty, a potentially severe condition. Sedentary behavior is a potentially modifiable factor and, therefore, the fact that over half of our population showed pre-frailty underscores the need for wider early identification and intervention efforts among older adults. Although pre-frail older adults will not necessarily become frail, they face the possibility that their condition may worsen. Indeed, around one-quarter^50^ to one-third^51^ of pre-frail adults can expect to transition to frailty within a year's time. This possibility of a worsening condition implies the need for timely management aimed at delaying or possibly preventing additional deterioration in pre-frail individuals and, hence, future functional losses. It is not clear whether specific interventions could prevent frailty in already-at-risk individuals, but studies seem to suggest that pre-frail older adults respond better to health interventions than do frail older adults.^52^^–^^54^

Importantly, previous studies demonstrated that more prolonged exposure to sedentary behavior is detrimentally associated not only with frailty, but also with higher levels of inflammatory markers^55^ and higher risk of cardiovascular diseases, cancer and all-cause mortality among older adults.^56^^,^^57^ There is still much to be understood about physiological maladaptation to sedentary behavior. However, finding interventions to reduce sedentary behavior effectively should be a key research priority for healthcare providers and policymakers. This is because sedentary individuals may experience a vicious cycle, in which loss of muscle mass and strength may lead to disability^4^ and disability boosts sedentariness.^58^ This, in turn, may perpetuate an elevated inflammatory load^55^ and promote subsequent additional loss of muscle mass.^18^ Despite the devastating consequences that loss of muscle mass, chronic inflammation and impaired physical function have on older adults, society and the economy, few interventions have been proven to be effective in counteracting age-related loss of muscle mass. While physical activity is known to be beneficial to the functioning of different systems in individuals with NCDs,^59^ and to be helpful in preventing functional declines, more intense and better targeted efforts are needed in order to disrupt the cycle of loss of muscle mass.

Due to the cross-sectional nature of the present study, our results should be interpreted with caution. However, this study was conducted on a representative sample of well-described community-dwelling older adults, and it underscores the notion that sedentary behavior can potentialize functional decline among both frail and pre-frail older adults. Therefore, future randomized controlled trials will be crucial in helping to find effective interventions, not only to improve adherence to regular physical activity, but also to help mitigate exposure to sedentary behavior and prevent further functional declines and adverse health outcomes.

## CONCLUSION

Frailty and pre-frailty were associated with sedentary behavior among these community-dwelling older adults. This study provides critical data upon which to base future strategies that would target diminution of sedentary behavior, so as to aid in prevention of pre-frailty and frailty among community-dwelling older adults.
